# Assessment of long-term alopecia after adjuvant taxane therapy for early breast cancer: a cross-sectional survey

**DOI:** 10.1007/s00520-025-09664-7

**Published:** 2025-06-23

**Authors:** Amy E. Smith, Michelle Harrison, Catriona McNeil, Jane Beith, Jennifer Lim

**Affiliations:** 1 Department of Medical Oncology, Chris O’Brien Lifehouse, 119-143 Missenden Rd, Camperdown 2050, Sydney, NSW Australia; 2 Department of Medicine, University of Sydney, Sydney, NSW Australia; 3 Department of Medicine, University of Notre Dame Australia, Sydney, NSW Australia

**Keywords:** Hair loss, Alopecia, Taxane, Chemotherapy, Toxicity, Survivorship

## Abstract

**Background:**

Alopecia is a distressing side-effect of taxane chemotherapy, and evidence suggests that docetaxel leads to chronic alopecia. We measured rates of satisfaction with hair regrowth among women who received adjuvant docetaxel compared with paclitaxel to identify a difference in outcomes.

**Methods:**

We identified adult female patients who received paclitaxel or docetaxel chemotherapy for early breast cancer from 2010 to 2015. They were screened to ensure they were alive, without metastatic relapse or a new cancer. Eligible participants were sent an introductory letter, consent, a Dermatology Life Quality Index (DLQI) questionnaire, a Visual Analogue Score (VAS) and the European Organisation for Research and Treatment of Cancer (EORTC) general and breast-specific quality of life. The primary outcomes were the DLQI: a 10-item questionnaire scored on a 0–3 scale (higher scores indicating distress and dissatisfaction) and the VAS (scored out of 4). Secondary outcomes were the global health status, function, symptom score and breast specific outcomes shown by the EORTC QLQ-C30 and EORTC QLQ-BR23.

**Results:**

There were 88 responders from 210 letters (response rate 42%); 38 (43%) had docetaxel and 50 (57%) had paclitaxel. They were aged 26–90 (median: 59). There was a significant difference in DLQI scores, with the docetaxel group having a higher median score (docetaxel: 4 vs paclitaxel: 1, *p* = 0.01). A significantly higher proportion of patients reported no effect of hair loss in the paclitaxel group (*p* = 0.02). Similarly, there was a significant difference in VAS scores with the docetaxel group having a higher median score (docetaxel: 1 vs. paclitaxel: 0.5, *p* = 0.002). Secondary outcomes did not reach statistical significance. There was no association with aromatase inhibitor exposure.

**Conclusions:**

Our study shows that adjuvant docetaxel chemotherapy is associated with statistically significant higher rates of dissatisfaction and chronic alopecia than paclitaxel chemotherapy. This supports literature and should be discussed prior to administering docetaxel regimens.

## Introduction

Early breast cancer is a common disease with around 20,000 new cases diagnosed in Australia in 2021 [1]. It is the most common cancer diagnosed in Australian women. Despite this, the mortality rate continues to drop, with 5-year survival improving from 72% in the 1970 s to 92% in 2010 s [2]. Detecting and treating breast cancer at the earliest possible stage allows for improved overall survival and less issues in the survivorship setting [1].

Taxane-containing chemotherapy regimens have been commonly used in the treatment of early breast cancer since the 1990 s [3, 4]. In early breast cancer, paclitaxel and docetaxel are the taxane agents utilised [3, 4]. These are well documented to improve overall survival and disease-free survival. A collaborative meta-analysis published in the Lancet in 2005 analysed 194 randomised controlled trials and found adjuvant poly-chemotherapy in women younger than 50 years to reduce the rate of annual breast cancer death by about 38% regardless of hormone or nodal status [4]. An updated Cochrane review that included 29 studies involving almost 42,000 women confirmed increased survival and time free of disease recurrence for women who received taxane-containing regimens in the adjuvant setting with the greatest benefit seen in lymph node-positive women [3].

While taxane-containing chemotherapy regimens are effective, they are not without side effects. Taxane-specific side effects which occur during treatment include peripheral neuropathy, myelosuppression, arthralgias, myalgias, pneumonitis and alopecia which can all impact a patient’s quality of life [5]. Often many of these adverse effects resolve with chemotherapy cessation, but many patients will experience persistent alopecia and peripheral neuropathy well into the survivorship setting [6].

Alopecia is a distressing side effect of chemotherapy for breast cancer patients [7]. Many patients who experience chemotherapy-induced-alopecia (CIA) report negative body image and decreased self-confidence [6]. This has been reported to cause up to 14% of patients to consider rejecting such life-saving treatments for fear of alopecia [6]. Research by Kang et al. [8] reported that patients treated with taxane-based regimens had up to eight times higher odds of permanent CIA 3 years after completion of chemotherapy compared to those who did not receive a taxane. Similarly, a case-series of 20 patients at 6 months following fluorouracil, epirubicin, cyclophosphamide and docetaxel (FEC-D) identified that 63% of women had moderate alopecia, 32% had intense alopecia and only 1 patient had minimal alopecia. These patients also had significantly higher Dermatology Life Quality Index (DLQI) scores indicating alopecia had a detrimental effect on their quality of life [9].

Our study aimed to measure rates of satisfaction with hair regrowth among women who received adjuvant docetaxel or paclitaxel and to compare these taxane agents to evaluate whether there were significant differences between these two taxane agents in the extent of alopecia induced to help guide future chemotherapy selection and inform chemotherapy counselling discussions for these patients.

## Methods

We conducted a single-centre, retrospective, cross-sectional questionnaire survey. Adult female breast cancer patients who were treated with adjuvant paclitaxel or docetaxel chemotherapy between January 2010 and December 2015 were identified through pharmacy records. Their medical records were screened to ensure they were still alive and did not have further metastatic relapse or a new cancer necessitating further treatment with chemotherapy. Written informed consent was obtained.

Eligible participants were mailed an introductory letter and surveys including a DLQI questionnaire, a Visual Analogue Score (VAS) which rated their overall satisfaction with their hair growth and the European Organisation for Research and Treatment of Cancer (EORTC) general (EORTC QLQ-C30) and breast-specific (EORTC QLQ-BR23) quality of life surveys. Patients were required to answer the survey in English.

Ethics approval was given by the human research ethics committee of the Royal Prince Alfred Hospital (HREC/16/RPAH/145) in accordance with the Declaration of Helsinki.

## Outcomes

The primary outcomes of interest for this study were the DLQI: a ten-item questionnaire and the VAS. Each question is measured on a 0–3 scale, with the overall score being a summation of the 10 items allowing a maximum total of 30 with higher scores indicating higher levels of distress and dissatisfaction.

Secondary outcomes of interest were the global health status, functioning status and symptom score as determined by the EORTC QLQ-C30 and the breast function and symptom score as assessed using the EORTC QLQ-BR23.

## Statistical analysis

Descriptive statistics were utilised for demographic and baseline characteristic data. Quality of life outcomes have been summarised as median (IQR) and differences between taxane chemotherapy groups were investigated using a Mann–Whitney *U*-test. The DLQI was split into categories using previously published cut-off points and analysed as an ordinal outcome using the same methodology. The statistical significance criterion was *p* < 0.05. Box plots were constructed to graphically visualise the differences.

## Results

In total, there were 88 responses from 210 invitations to participate in this study which gave a response rate of 42%. Of these, 38 patients (43%) had received docetaxel, and 50 patients (57%) had received paclitaxel. Respondents were aged between 26 and 90 (median, 59; IQR, 52 to 68), and the time since diagnosis ranged from 1 to 10 years (median, 3.8 years; IQR, 3 to 6 years). A total of 50 women who responded to the survey invitation (57%) were born in Australia and New Zealand, and 31 (43%) were born overseas with Asia, UK and Middle East being common places of birth outside of Australia and New Zealand. Of the 50 women born in Australia, 2 (4%) identified as Aboriginal or Torres Strait Islander (Table 1).

**Table 1:** Baseline characteristics of women who responded to the survey invitation

		Paclitaxel (%)	Docetaxel (%)	Total patient number (%)
Age (at diagnosis)	≥ 50 years	37 (62)	23 (38)	60 (68)
	< 50 years	14 (50)	14 (50)	28 (32)
Region of birth	Australia + NZ	31 (62)	19 (38)	50 (57)
	Asia	4 (36)	7 (64)	11 (13)
	European	5 (50)	5 (50)	10 (11)
	UK	4 (44)	5 (56)	9 (10)
	Middle East	5 (83)	1 (17)	6 (7)
	Other	1 (50)	1 (50)	2 (2)
Cultural group	Australian Aboriginal or Torres Strait Islander	1 (50)	1 (50)	2 (4)
	Unknown	50 (58)	36 (42)	86
Chemotherapy regimen	AC-T	38 (100)	0 (0)	38 (43)
	FEC-D	0 (0)	24 (100)	24 (27)
	TC	0 (0)	14 (100)	14 (16)
	EC-T	6 (100)	0 (0)	6 (7)
	Other	4 (67)	2 (33)	6 (7)
Nodal status	N0	19 (51)	18 (49)	37 (42)
	N1	24 (56)	19 (44)	43 (49)
	N2	2 (50)	2 (50)	4 (4.5)
	N3	2 (50)	2 (50)	4 (4.5)
Comorbidity	Thyroid disease	6 (75)	2 (25)	8 (9)
	Scalp disorder	2 (50)	2 (50)	4 (4.5)
	Anaemia	4 (57)	3 (43)	7 (8)

**Fig. 1:**
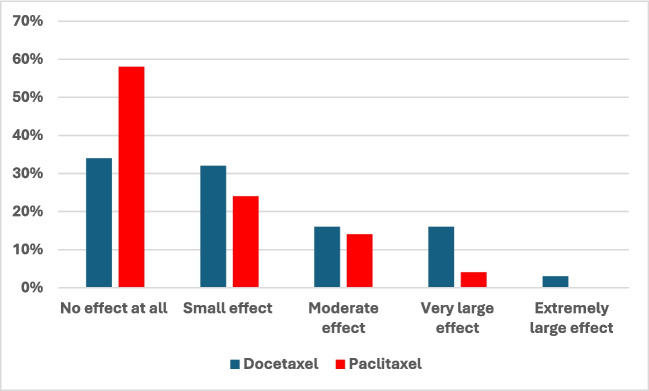
Effects of hair loss on patient’s quality of life by taxane-chemotherapy group

The DLQI scores reported by survey responders are shown visually in Fig. 1 and summarised in Table 2. A higher proportion of patients in the paclitaxel group compared to the docetaxel group reported that hair loss was having no effect on their lives, and conversely, a significantly higher proportion of patients in the docetaxel group experienced moderate or very large effects (*p* = 0.02). Overall, we identified a significant difference between the median scores (docetaxel: 4 vs paclitaxel: 1, *p* = 0.01). Similarly, there was a significant difference in the VAS scores reported by patients with the docetaxel score having a higher median value (Docetaxel: 1 vs paclitaxel:0.5, *p* = 0.002).

**Table 2:** DLQI and VAS scores summarised as continuous and ordinal variables by taxane-chemotherapy group

	Docetaxel (*n* = 38)	Paclitaxel (*n* = 50)	*p*-value
VAS	1 (1–3)	0.5 (0–1)	0.002
DLQI Score			
Median (IQR)	4 (1–10)	1 (0–5)	0.01
Effect of patient’s life:			0.09
No effect at all	13 (34%)	29 (58%)	
Small effect	12 (32%)	12 (24%)	
Moderate effect	6 (16%)	7 (14%)	
Very large effect	6 (16%)	2 (4%)	
Extremely large effect	1 (3%)	0	

DLQI score interpretation: 0–1 no effect on patients’ life, 2–5 small effect on patients’ life, 6–10 moderate effect on patients’ life, 11–20 very large effect on patients’ life, 21–30 extremely large effect on patients’ life.

The secondary quality of life outcomes are summarised in Table 3 according to specific taxane-chemotherapy group. Overall, there was a trend for patients in the paclitaxel chemotherapy group to report a higher quality of life, but this did not reach statistical significance (*p* = 0.06).

**Table 3:** Summary of QoL outcomes by taxane-chemotherapy groups

	Docetaxel (*n* = 30)	Paclitaxel (*n* = 34)	*p*-value
	Median (IQR)	Median (IQR)	
EORTC			
Global health status	75 (58–75)	83 (67–83)	0.06
Functional status	84 (73–91)	86 (73–91)	0.61
Symptom score	14 (7–26)	18 (3–26)	0.99
EORTC-BR23			
Functional status	45 (38–71)	47 (35–62)	0.57
Symptom status	17 (12–29)	15 (9–31)	0.79

## Discussion

**Fig. 2:**
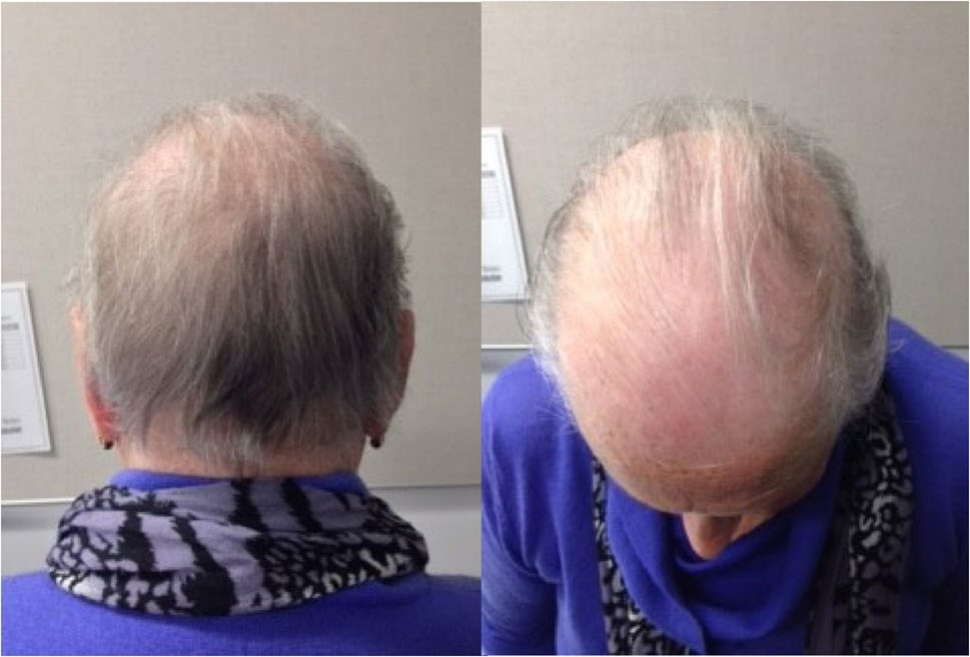
Permanent alopecia from docetaxel chemotherapy

Persistent and permanent CIA (Fig. 2) is an important issue that requires consideration at the time of prescription of potentially curative chemotherapy regimens. Often the prime focus at initial diagnosis is on cure. However, with the increased rates of survival from early breast cancer and the increasing understanding of survivorship, more consideration needs to be given to chemotherapy choice and potential long-term side effects.

We report the first cohort of patients with quality-of-life impacts from CIA from both paclitaxel- and docetaxel-containing chemotherapy regimens for the treatment of early-stage breast cancer. While docetaxel has a well-reported association with CIA, prior to this study, only a small number of case reports were available for paclitaxel. In our study, a higher proportion of patients in the paclitaxel group compared to the docetaxel group reported that hair loss was having no effect on their lives. Conversely, a higher proportion of patients in the docetaxel group experienced very large negative effect on their quality of life. While objective alopecia is important, arguably, the more important finding is the impact this has on survivorship as not all patients will report the same negative quality of life impact for the same degree of alopecia.

There are several well-described treatment options for regrowth of hair for those who have experienced CIA with varying levels of evidence. However, the only readily available preventative option is scalp cooling. A retrospective review of scalp cooling found that younger patients with private health insurance were more likely to opt for scalp cooling [10]. However, in the public Australian health care setting, scalp cooling requires more resources and is not offered at every public cancer centre. This study was completed prior to availability of scalp cooling at our centre.

A growing area of complexity and interest is delivery of oncological care in culturally appropriate ways for patients who are culturally and linguistically diverse (CALD). Work by Pleasant et al. [11] focussed on the issue of CIA for Black women, highlighting the cultural importance hair can have in different CALD groups. For these women, hair has significant historical, social, political and cultural implications. Additionally, clinical trials that have tested scalp cooling often have not included many Black women who typically have hair that differs in texture and thickness from other women, and studies have not been powered to detect differences of alopecia between CALD groups [11].

While our study did not ask participants which (if any) cultural group they belonged to, or identified with, 44% of our patients were not born in Australia. Of the patients born in Australia, 4% identified as Aboriginal and Torres Strait Islander. A literature review of health-related quality of life issues for Aboriginal and Torres Strait Islander patients experiencing cancer reported several side effects, including hair loss, which made them feel sicker rather that healthier [12].

These results changed practice at our institution which is why data collection was stopped at 2015. The results of this survey helped influence decision making regarding choice of poly-chemotherapy regimen with sequential taxane chemotherapy away from docetaxel-containing regimens, particularly FEC-D in favour of AC-T for higher risk patients.

## Limitations

This study is limited by several factors including the potential for recall bias due to the retrospective nature and selection bias given the low response rate (42%) and subsequently limited sample size. The voluntary and retrospective nature of the study would suggest that patients who were impacted by CIA were more likely to respond. Pleasingly, despite this, there was equal representation between both the paclitaxel and the treatment docetaxel groups.

Alopecia was not measured objectively (with photographs) but depended on a subjective response. Since the impact of alopecia is related to the patient’s perceived quality of life, it could be argued that subjective assessment is the most relevant outcome for this issue.

Another limitation is that this study did not adjust for other causes of alopecia (such as menopausal status, age, pregnancy and hypothyroidism).

Finally, this study focused on only one area of chronic toxicity (alopecia) and did not evaluate neuropathy which can also be a functionally debilitating permanent toxicity and survivorship issue caused by taxane-based chemotherapy.

## Conclusion

Our cross-sectional retrospective study is the largest to our knowledge looking at quality of life outcome measures for patients who received adjuvant paclitaxel or docetaxel chemotherapy for early breast cancer. Our study suggests that adjuvant docetaxel chemotherapy is associated with higher rates of dissatisfaction due to persistent or permanent CIA than adjuvant paclitaxel chemotherapy. This supports previous literature identifying persistent and permanent CIA as a survivorship issue and that patients should be adequately counselled on this as part of the consent process for chemotherapy. Greater efforts are needed to increase access to scalp-cooling as a means to reduce the impact CIA has in the survivorship setting. Future studies could examine the effectiveness of scalp cooling with paclitaxel or docetaxel. Finally, further work looking at the experience of CALD women and alopecia will be increasingly useful in not only treatment counselling but also in the survivorship setting.

## Data Availability

Data is provided within the manuscript.
